# A model based on artificial intelligence for the prediction, prevention and patient-centred approach for non-communicable diseases related to metabolic syndrome

**DOI:** 10.1093/eurpub/ckaf098

**Published:** 2025-07-03

**Authors:** Alejandro Clarós, Andreea Ciudin, Jordi Muria, Lluis Llull, Jose Àngel Mola, Martí Pons, Javier Castán, Juan Carlos Cruz, Rafael Simó

**Affiliations:** Higia.ai, Barcelona, Spain; Diabetes and Metabolism Research Group, VHIR, Endocrinology Department, Vall d’Hebron University Hospital, Autonomous University Barcelona, Barcelona, Spain; CIBERDEM (Instituto de Salud Carlos III), Madrid, Spain; Higia.ai, Barcelona, Spain; Higia.ai, Barcelona, Spain; Higia.ai, Barcelona, Spain; Higia.ai, Barcelona, Spain; Higia.ai, Barcelona, Spain; Higia.ai, Barcelona, Spain; Diabetes and Metabolism Research Group, VHIR, Endocrinology Department, Vall d’Hebron University Hospital, Autonomous University Barcelona, Barcelona, Spain; CIBERDEM (Instituto de Salud Carlos III), Madrid, Spain

## Abstract

Metabolic syndrome (MetS) is related to non-communicable diseases (NCDs) such as type 2 diabetes (T2D), metabolic-associated steatotic liver disease (MASLD), atherogenic dyslipidaemia (ATD), and chronic kidney disease (CKD). The absence of reliable tools for early diagnosis and risk stratification leads to delayed detection, preventable hospitalizations, and increased healthcare costs. This study evaluates the impact of Transformer-based artificial intelligence (AI) model in predicting and managing MetS-related NCDs compared to classical machine learning models. Electronical medical data registered in the MIMIC-IV v2.2database from 183 958 patients with at least two recorded medical visits were analysed. A two-stage AI approach was implemented: (1) pretraining on 60% of the dataset to capture disease progression patterns, and (2) fine-tuning on the remaining 40% for disease-specific predictions. Transformer-based models was compared with traditional machine learning approaches (Random Forest and Linear Support Vector Classifier [SVC]), evaluating predictive performance through AUC and F1-score. The Transformer-based model significantly outperformed classical models, achieving higher AUC values across all diseases. It also identified a substantial number of undiagnosed cases compared to documented diagnoses fold increase for CKD 2.58, T2D 0.78, dyslipidaemia 1.89, hypertension 3.33, MASLD 5.78, and obesity 4.07. Diagnosis delays ranged from 90 to 500 days, with 35% of missed intervention opportunities occurring within the first five appointments. These delays correlated with an 84% increase in hospitalizations and a 69% rise in medical procedures. This study demonstrates that Transformer-based AI models offer superior predictive accuracy over traditional methods by capturing complex temporal disease patterns. Their integration into clinical workflows and public health strategies could enable scalable, proactive MetS management, reducing undiagnosed cases, optimizing resource allocation, and improving population health outcomes.

## Introduction

Metabolic syndrome (MetS) is defined by a cluster of comorbid diseases associated with insulin resistance (IR) that increase the risk of cardiovascular morbi-mortality [1]. The diagnosis of MetS is currently based on one of two main criteria (abdominal obesity or global obesity) plus two of three additional diagnostic criteria (glucose metabolism impairment, high levels of non-HDL cholesterol, arterial hypertension [HTA]), with no other factors considered. Patients with MetS are at high risk of developing non-communicable diseases (NCDs) such as type 2 diabetes (T2D), metabolic-associated steatotic liver disease (MASLD), atherogenic dyslipidaemia (ATD), chronic kidney disease (CKD), and heart failure (HF) [2]. MetS prevalence has been estimated to be about 24.3% in a study performed in 2016 [3] on 34 821 subjects from 10 European countries, based on the classical criteria for MetS [1].

Recent research has opened interesting avenues for enhancing MetS risk stratification, and thus prevention, namely findings on: (a) the relevance of genetic factors, with up to 50% of MetS being inherited [4], (b) MetS clustering in adipose, lipid, or metabolic-based, suggesting that the mechanisms of progression form MetS to T2D, MASLD, CKD, ATD, or HF are not the same [5], and (c) social and environmental factors, including geographic, racial, ethnic origin, and person related [6]. Thus, it is clear that MetS is characterized by a complex interplay of various pathologies and factors, and relying solely on few clinical biomarkers for prediction or diagnosis is limiting our understanding of its holistic nature. Currently, these factors mentioned above are not taken into account for diagnosis of MetS and/or evaluation and risk stratification for MetS-related NCDs.

At present, there are no reliable data regarding the real prevalence of MetS across Europe, especially in the context of obesity pandemics, which could further increase the MetS prevalence at all ages. Since 1980, the global prevalence of obesity, the main criteria for MetS, has increased above 27% in adulthood and 47% in childhood [7]. Furthermore, recently the European Association for the Study of Obesity (EASO) published a new framework for the diagnosis of obesity, including body mass index (BMI) > 25kg/m^2^ with waist-to-height ratio (WtHr)  > 0.5 that will potentially significant impact in the real prevalence of MetS [8]. Additionally, the WtHr was proven to be strongly associated with NCD rather than BMI itself [9]. At present, there is no tool that allows us to perform a precise clustering and characterization of MetS, based on new diagnosis methods and related factors, as explained above, and to assess the risk of developing MetS-related NCD. In this context, MetS-related diseases are reaching a heavy burden for individuals, society, and economy, through high public health costs due to its complications. The economic impact of this lack of public health policies is huge: about 7% of the national budgets across the EU is spent on NCD associated with obesity every year [10]. Recent data showed the estimation of prevalence of obesity worldwide both in adults [11] and in children [12], which will reach more than 50% in all countries. Furthermore, to date, no country has managed to reach effective intervention for overweight and obesity and implicitly for MetS and its related complications.

Despite this evidence and heterogeneous features of MetS and related NCDs across countries, currently, a ‘one-size-fits-all’ reactive strategy for the management of MetS is implemented. The current strategy is based on treating the complications and is mainly due to the lack of reliable tools to identify in a timely manner those subjects at risk, as well as to predict the MetS-related NCD development. This reactive strategy is largely inefficient and has a negative societal and economic impact due to the delay in the diagnosis as has been recently reported for diabetes [13], thus increasing the risk of specific complications. Similar results have been reported for CKD [14], HTA [15], MASLD [16], and HF [17], showing an overall increase in all-cause morbidity and mortality.

Acknowledging this challenge, a multi-dimensional and proactive approach is needed. This multi-dimensional approach requires vast amounts of multi-domain data, as well as data management and an artificial intelligence (AI) architecture capable of connecting all this knowledge to work towards a single objective that puts the patient at the centre. This study proposes the creation of solutions beyond the ‘state-of-the art’ based on AI capable of predicting the onset of MetS and related complications. AI-based tools can be deployed in various healthcare settings, including primary care clinics and remote telemedicine platforms, potentially making MetS prevention and/or diagnosis more accessible to a broader population. Moreover, the scalability of AI allows it to handle large datasets and support population-wide preventive initiatives. From a technical perspective, traditional machine learning models, such as Random Forest and LinearSVC, have been widely applied in healthcare for predictive tasks due to their simplicity and interpretability. Random Forest [18], with its capacity to handle overfitting and categorical data, and LinearSVC [19], a linear classification model, are two of the most commonly used approaches in clinical settings. However, these models often struggle with multi-dimensional, longitudinal health data, limiting their effectiveness in capturing complex patient trajectories [20]. To overcome these limitations, recent advancements in Transformer-based models have emerged [21]. These models offer superior versatility in processing sequential, high-dimensional patient data, allowing for more accurate predictions of disease onset.

On these bases, the main aim was to compare the predictive performance of AI Transformer-based approaches with the traditional models in predicting the onset of MetS and related NCD, as a ‘proof-of concept’ study.

## Methods

### Dataset and patient cohort

This study uses the MIMIC-IV database v2.2 [22, 23], which includes de-identified health records of over 299 712 patients admitted to the Beth Israel Deaconess Medical Center, in Boston (USA) between 2008 and 2022, with 3 223 469 hospital encounters. The dataset covers various clinical variables, such as demographics, anthropometric measurements, laboratory tests, medications, symptoms, and registered diagnoses. For this study, we filtered the cohort to include individuals with at least two distinct medical visits, with at least 1 year between them, resulting in a final cohort of 183 958. This threshold ensured sufficient follow-up time for assessing the progression and prediction of MetS-related NCD.

No additional clinical or demographic filters were applied to maximize the heterogeneity of the dataset, reflecting a broad patient population. This approach aimed to increase the generalizability of the model across diverse clinical profiles and minimize selection bias. However, there still is some selection bias, particularly due to the hospital-based nature of the dataset. As MIMIC-IV is derived from an academic hospital, the cohort likely overrepresents individuals with more severe or advanced disease stages, while underrepresenting healthier individuals or those visiting primary care centres. Additionally, the population reflects the healthcare patterns of an urban US hospital, which may limit the generalizability of the finding to non-US or low-resource settings. Despite these limitations, the broad inclusion strategy increases the likelihood of capturing heterogeneous disease progressions across different patient subgroups. Moreover, this approach allows for potential transfer learning, where the resultant model can be fine-tuned on other region-specific datasets while retaining its core predictive capabilities, thereby increasing its adaptability and effectiveness in diverse healthcare settings.

### Modelling approach

To effectively model the complex interplay between MetS-related NCD, we employed a two-stage approach of general pretraining followed by disease-specific fine-tuning, for two reasons. First, all the NCDs we aimed to model—T2D, CKD, HTA, HF, and CVD—share a common origin in IR and MetS, with overlapping risk factors, clinical presentations, and biomarkers, as explained in the introduction. By pretraining a general model across all patient data, we ensured that the model captured the underlying correlations and shared biological pathways between these conditions. This pretraining step allowed the model to learn the complex interactions between biomarkers such as blood glucose, creatinine, lipid levels, BMI, and blood pressure, as well as the temporal dynamics of disease progression. Second, this approach allows for model transferability. By training a generalized model on a large and diverse dataset such as MIMIC, we were able to create a model that could be extrapolated to diverse disease contexts. The main advantage is when applying the model to other datasets or clinical environments where fewer labelled samples may be available for a specific disease. The general pretrained model can be fine-tuned on smaller datasets, still achieving robust predictive performance by leveraging the knowledge gained during pre-training. This flexibility is crucial for scaling the model to different populations and healthcare systems where comprehensive data might not be available.

The hyperparameter optimization process was performed separately for each stage. During pretraining, we used a combination of grid search and manual tuning based on validation performance optimizing hyperparameters such as learning rate, batch size, dropout rate, and model architecture.

Given the natural imbalance of NCD prevalence in the population, we applied class imbalance to the loss function during fine-tuning. This approach assigned higher penalties to misclassified positive classes, mitigating the risk of biased predictions. Moreover, model evaluation was based on multiple metrics, mainly AUC and F1-score, to ensure performance was not driven solely by majority classes.

Success thresholds were predefined based on literature benchmarks, with AUC > 0.7 considered acceptable predictive performance. These predefined criteria ensured that model performance was both statistically robust and clinically meaningful.

### Data preprocessing and cleaning

The dataset underwent systematic pre-processing procedure to ensure data quality and consistency:

- Handling missing values: null values for critical features, such as blood pressure, weight, and height, were addressed through imputation or removal of implausible values (e.g. extreme outliers in height and weight were corrected or excluded).
- Feature engineering: additional variables were derived from the existing dataset, including calculating BMI from height and weight, and categorizing laboratory results into clinically relevant bins (e.g. normal, high, critical).
- Text-based input and sequence generation: text input allowed the generation of sequences, facilitating the elimination of missing data and reducing variability in visit counts and patient values.
- Synthetic patient diagnostics: patient data were augmented with simulated diagnoses, improving model robustness in scenarios with limited real-world data.

### Pre-training on patient data

We utilized a Transformer-based model to pretrain on patient visit sequences, leveraging this architecture to capture complex medical patterns. For this phase, 60% of the MIMIC dataset was used for pretraining, while the remaining 40% was set aside for fine-tuning.

During training, we applied strategies to make the model learn to predict missing pieces of patient visit data based on the surrounding information. This approach enabled it to recognize relationships between key medical events such as lab results, treatments, and diagnoses, helping it capture underlying patterns. Additionally, the model was trained to understand the logical order of patient visits, allowing it to track the progression of diseases over time and improve its understanding of how conditions evolve across multiple visits.

Throughout pretraining, patient visit sequences, represented as structured tokens, were processed across multiple epochs. To ensure optimal performance, we applied early stopping to prevent overfitting and used weight decay to fine-tune the learning rates, further enhancing the model’s ability to generalize effectively.

### Fine-tuning disease-specific models

After pretraining, we fine-tuned the model separately for each target disease. These fine-tuned models included predictions for conditions such as T2D, CKD, hypertension, ATD, and obesity. The fine-tuning process tailored the pretrained model’s general understanding to the specific nuances of each disease, improving predictive accuracy in each domain.

To ensure robustness, we used stratified 10-fold cross-validation across the dataset, optimizing hyperparameters such as learning rates and batch sizes for each disease model. This stratification ensured that the models were exposed to diverse subsets of the data, enhancing their ability to generalize to new, unseen cases.

- Training and validation data: the portion of the MIMIC dataset reserved for fine-tuning was divided into 70% for training, 20% for validation, and 10% for testing. This allocation ensured a sufficiently large training set while retaining enough data for robust validation. The split enabled the model to learn from a diverse range of patient data and evaluate its generalization to unseen validation data.
- Testing: after fine-tuning, the models were tested on an entirely unseen subset of the MIMIC dataset. No data augmentation or synthetic data were used, ensuring that the model’s performance was evaluated under real-world conditions, enhancing its clinical applicability.

### System performance in detecting undiagnosed patients

The system was designed to detect patients who, despite not having a formal diagnosis, exhibit signs of disease based on their lab results and physiological features. This was accomplished through a two-layer analysis that highlights discrepancies between clinical signs and formal diagnoses:

- Verification of existing diagnoses: this step ensured that patients already diagnosed were filtered from further analysis.
- Identification of undiagnosed cases with lab and physiological indicators: this analysis identified patients with abnormal lab results (e.g. elevated glucose, abnormal creatinine) that suggest disease presence but lack a formal diagnosis. The evaluation was conducted according to the WHO guidelines for clinical and laboratory diagnoses, ensuring that the identified indicators align with globally recognized diagnostic criteria.

### Evaluation metrics

The following metrics were used to evaluate the performance of each disease-specific model:

- Area under the curve (AUC): the primary metric for distinguishing between patients with and without the target disease.
- Precision, recall, and F1 score: used to balance the model’s performance in terms of true positives and false positives, providing insights into the trade-offs between precision and recall.

## Results

### Pre-training model results

The pre-training phase yielded favourable results, with a training loss of 0.074 and an evaluation loss of 0.0869 after a few epochs. The masked language modelling (MLM) and next sentence prediction (NSP) tasks enabled the model to understand both the context and the temporal progression of patient health data.

### Fine-tuned model results

The fine-tuning phase aimed to assess the predictive performance of the Transformer-based approach against classical machine learning models across multiple NCDs. Fine-tuning was performed by coupling the Transformer embeddings with classical classifiers, leveraging the pre-trained model’s capacity to encode rich temporal patterns from patient health records. A grid search was conducted to identify the optimal classifier for downstream tasks, with Linear SVC emerging as the best-performing model.

Table 1 presents a comparative analysis of AUC values for the different diseases, illustrating the superior performance of the Transformer-based approach compared to traditional machine learning models such as Random Forest and Linear SVC. The Transformer Approach + Linear SVC consistently achieved higher AUC values across most diseases, indicating enhanced predictive accuracy.

**Table 1.** Models performance comparison

Disease	Transformers + LinearSVC	Random Forest	LinearSVC
	(AUC)	(AUC)	(AUC)
Type 2 diabetes (T2D)	0.90	0.58	0.57
Chronic kidney disease (CKD)	0.91	0.69	0.68
Arterial hypertension (HTA)	0.80	0.66	0.63
Atherogenic dyslipidaemia (ATD)	0.79	0.66	0.65
Obesity	0.79	0.70	0.68
Metabolic-associated steatotic liver disease (MASLD)	0.90	0.58	0.60

All models were trained on the same dataset, ensuring a methodologically consistent comparison. The predictions generated by these models were designed to forecast disease onset up to 1 year in advance, demonstrating the potential of Transformer-based approaches in predictive healthcare modeling.

The superior performance of Transformer-based models compared to classical machine learning models can be attributed to their ability to capture temporal dependencies and complex interactions in patient health records, thanks to their attention mechanisms. Traditional models, such as Random Forest and Linear SVC, rely on pre-aggregated features per patient (e.g. summary statistics like minimum, maximum, or average values), which reduces the accuracy of data representation and limits the model’s ability to leverage all available information. Attention mechanisms in Transformer-based models effectively address these shortcomings.

In our study, classical models analyse at most 65 aggregated variables per patient, including ‘mean glucose’, ‘mean hemoglobin A1c’, and ‘diabetes diagnosed’. For patients without certain measurements, missing values techniques were applied by imputing the median value for continuous variables and the most frequent value for categorical variables. This approach ensured a consistent representation of patient data across all samples, at the cost of potential information loss. In contrast, the Transformer-based approach processes all available patient records without aggregation, encoding as many features as the patient has recorded. For example, a patient with 12 visits could yield approximately 300 features, offering a significantly richer and more accurate representation of the clinical history.

Furthermore, Transformer architectures are designed to handle a large number of trainable parameters while preserving the temporal order of patient data. Table 2 compares the number of theoretic trainable parameters between the Transformer-based approach and classical models, emphasizing the increased capacity of Transformers to process complex datasets.

**Table 2.** Trainable parameters comparison

Model	Parameters
Transformer + LinearSVC approach	> 100.000.000
Linear SVC	< 1.000
Logistic Regression	< 1.000
Random Forest	< 250.000

**Figure 1.**
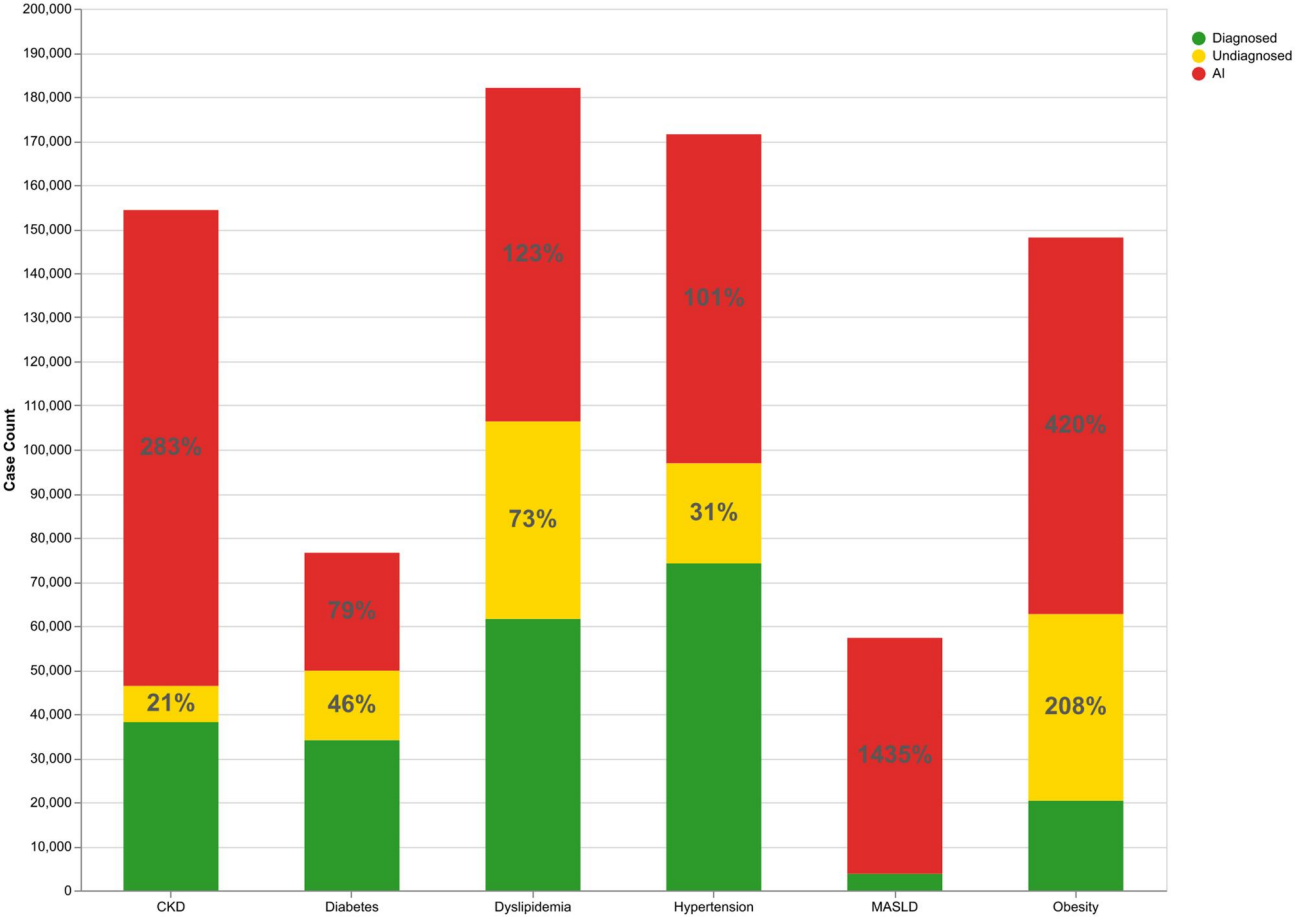
Diagnosed, undiagnosed and at-risk population in 2021, based on the MIMIC dataset.

Beyond their superior predictive performance, Transformer-based models offer additional insights by generating patient-specific risk scores that allow us to identify which portions of the population are at risk of developing MetS-related NCD. These risk scores enable us to analyse the population both retrospectively and prospectively—understanding past trends while predicting future risk—so that we can pinpoint individuals most vulnerable to these diseases. The proportion of population undiagnosed of the mentioned NCD or at high risk to acquired them is represented in Fig. 1. The chart reveals significant gaps between diagnosed cases and the broader population at risk, indicating a large proportion of undiagnosed or at-risk individuals for each disease.

**Figure 2.**
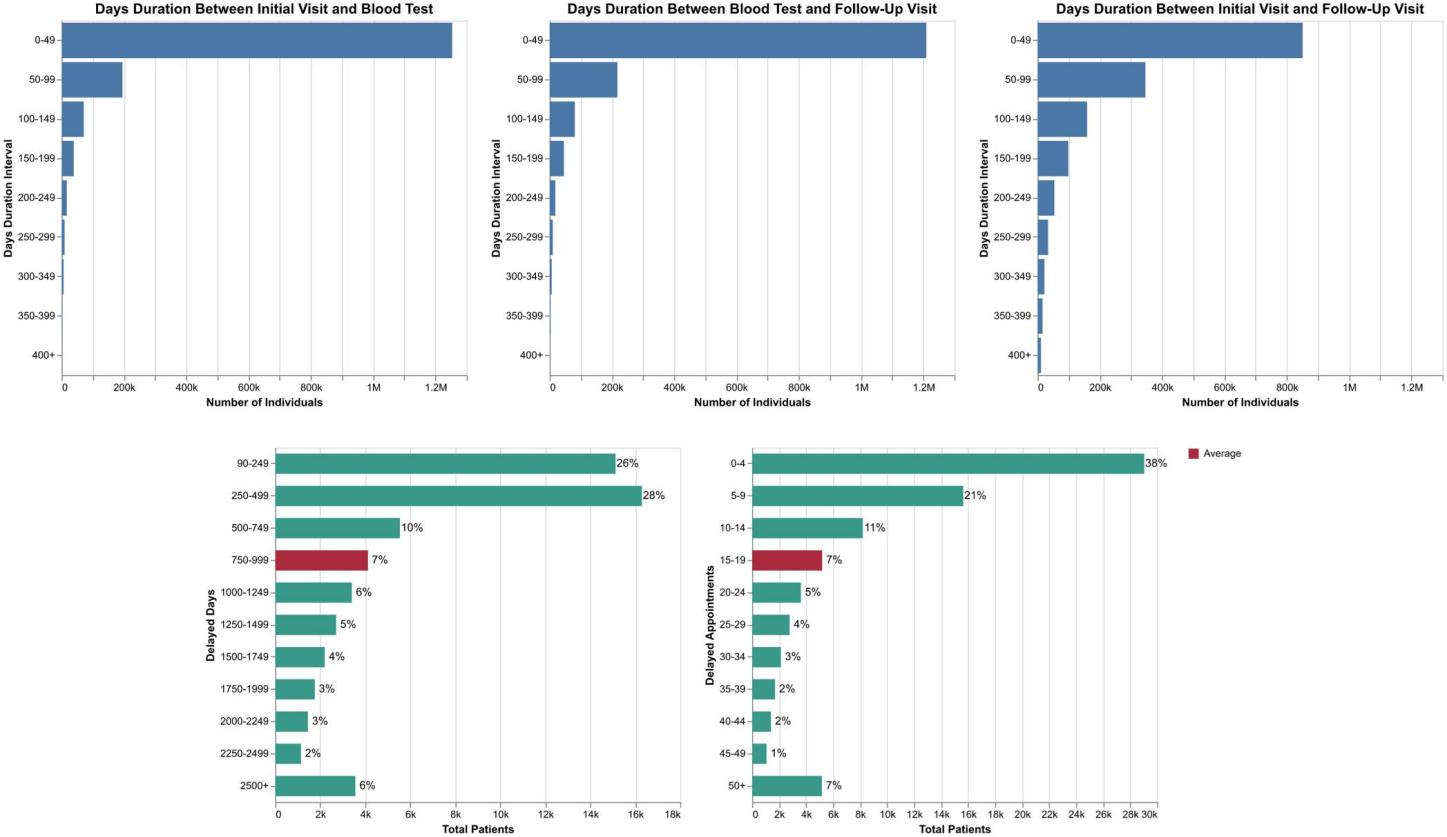
Number of days between key stages of diagnosis/impact of diagnostic delays and missed follow-up appointments.

Moreover, using the MIMIC dataset the model evaluated the effect of diagnostic delays on patient outcomes. The system identified patterns where earlier detection of chronic conditions could have mitigated the need for more advanced follow-up care. The time between a patient’s initial visit, lab results, and follow-up appointments was reviewed. Figure 2A shows the number of days between key stages of diagnosis and Fig. 2B shows the statistics calculated for all diseases considered in this paper. These data reveal that nearly 50% of patients experienced delays ranging from 90 to 500 days. Furthermore, 35% of missed opportunities for intervention occurred within the first 0–5 delayed appointments. Data for each NCD separately analysed are shown in Annex-Supplementary Material.

**Figure 3.**
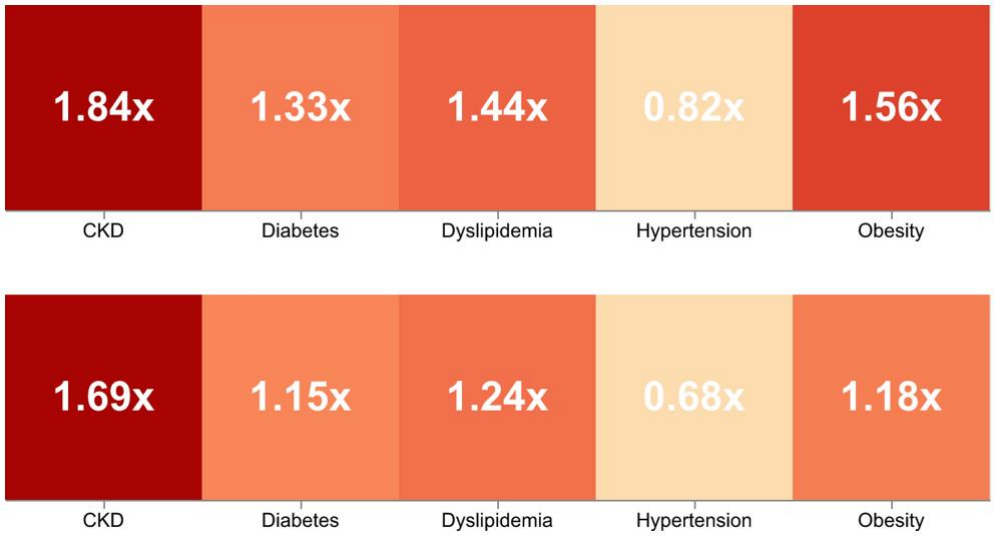
Comparing hospitalization rates over 5 years: delayed diagnosis vs timely diagnosis/comparing procedure rates over 5 years: delayed diagnosis vs timely diagnosis.

The delay in diagnosis significantly impacted the patient’s outcomes. When comparing subjects in whom timely diagnoses were performed with those with delayed diagnoses, we found that the latter experienced significantly more hospitalizations and procedures over a 5-year period. Specifically, for CKD, a delayed diagnosis resulted in up to 84% more hospitalizations and up to 69% more procedures, as shown in Fig. 3A and B, respectively.

### Ethical considerations

This study utilized the MIMIC-IV v2.2 database, a publicly available and de-identified dataset comprising electronic health records from the Beth Israel Deaconess Medical Center. The de-identification process ensures compliance with the Health Insurance Portability and Accountability Act (HIPAA), thereby waiving the need for individual informed consent.

Access to the dataset was granted following completion of the required training and adherence to the PhysioNet Credentialed Health Data Use Agreement (https://physionet.org/content/mimiciii/view-dua/1.4/), which outlines the ethical use and handling of sensitive health information.

To enhance model robustness and protect privacy, synthetic patient data were generated for certain analyses. While synthetic data can mitigate privacy risks, it does not eliminate potential biases. To address this, synthetic data generation followed strict validation procedures to ensure consistency with real-world clinical patterns.

Researchers are reminded that, despite the de-identified nature of the data, ethical obligations persist in terms of data handling and analysis. It is essential to ensure that findings derived from such datasets do not inadvertently compromise patient confidentiality or lead to misuse of information.

While the MIMIC-IV dataset offers a rich source of clinical information, it predominantly represents patients from a single urban academic medical centre in the USA. This demographic concentration may limit the generalizability of findings to broader or different populations. Researchers should consider this context when interpreting results and acknowledge potential biases stemming from the dataset’s composition.

## Discussion

Current state of the art on AI for prediction of MetS and related comorbidities predominantly is based on forecasting the individual NCD, such as T2D [24] or MASLD [25] in a reactive manner.

Some other studies focused on prediction of MetS based on ‘big-data’ have been focused on single risk factors, such as genetics, ethnicity, or metabolomics [26]. Therefore, current strategies do not provide a holistic proactive solution for the management of the metabolic NCDs.

In the present study, we have evaluated a digital tool that permits characterization of MetS risk and risk of developing MetS-related NCDs in a personalized manner, as well as allowed a characterization of the current approach in the community for MetS-related NCDs. The proposed model was able to identify a significant proportion of new cases that were overlooked in the medical records, for each one of the NCDs that was analysed and showed superiority by comparison with traditional AI strategies. This finding demonstrates the urgent need for enhanced screening, early detection, and proactive management to close these gaps in care and improve health outcomes for at-risk populations. Furthermore, the model showed that a significant delay in the diagnosis in all the evaluated NCDs occurs, as well as a significant number of missed opportunities for being diagnosed during routine visits to the medical centre.

Delays in diagnosing MetS-related NCDs drastically impact patient health outcomes, leading to increased morbidity, disease progression, and premature mortality. As a reference, our study found that diagnostic delays were associated with an 84% increase in hospitalizations and a 69% increase in procedures for CKD cases, demonstrating the severe consequences of a reactive healthcare model. Furthermore, 35% of cases could have been diagnosed within the first five visits, highlighting missed opportunities for early intervention. By the time diseases such as CKD or MASLD are detected, patients often require intensive treatments, increasing healthcare costs, and straining medical resources.

These delays disproportionately impact vulnerable populations, exacerbating health inequities. Current diagnostic frameworks fail to incorporate genetic, socioeconomic, and lifestyle factors, limiting their ability to effectively stratify at-risk individuals. Without a proactive strategy, healthcare systems continue to operate inefficiently, leading to higher costs and poorer long-term health outcomes. Our study provided evidence that the delay in the diagnosis had a significant impact in patients’ outcomes, in particular hospitalizations and number of procedures, resulting in a higher utilization of healthcare resources, increasing costs related to this reactive and inefficient current approach, commonly implemented in the daily clinical activity.

In Europe, there are no specific programmes for MetS diagnosis, prevention, and treatment. In some countries, public policy programmes for obesity prevention are starting to develop, but they are far from being holistic, encompassing the NCD, patient centred, timely optimized, and/or efficiently implementable on a large scale. Current approaches are based on strategies for prevention using life-style changes (mainly diets and physical exercise recommended usually in face-to-face on-site interaction between individuals and HCPs, in an ‘one-size-fits-all’ manner). Furthermore, the economic impact of this lack of public health policies is huge: about 7% of the national budgets across the EU is spent on NCD associated with obesity every year [10]. These data highlight the need for updated surveillance strategies aligned with the European Commission’s NCD prevention initiatives and the WHO European Regional Obesity Report, which emphasize early detection and risk stratification for metabolic diseases.

Additionally, the diagnosis approach is based on delayed reactive strategy focused on the established complications. As a consequence, the prevalence of MetS and related NCD are growing alarmingly, as well as the associated costs in all the healthcare Systems. In order to change the paradigm from ‘reactive’ to ‘proactive’, a multi-dimensional strategy is required. This implies vast amounts of multi-domain data, as well as data management that can be efficiently performed by an AI architecture. The absence of harmonized MetS monitoring systems in Europe underscores the urgency of AI-driven real-time epidemiological surveillance, aligning with the European Health Data Space (EHDS) initiative to enhance cross-border health data interoperability. Implementing AI-driven predictive analytics within national healthcare policies could support the European Strategy for Data, allowing healthcare systems to transition towards more data-driven, personalized medicine approaches.

Healthcare is starting to use AI and associated technologies, which are becoming more and more common in industry and society. AI methodologies proposed so far are rooted in traditional classification and clustering techniques, encompassing Decision Trees, Naive Bayesian, Support Vector Machines, Random Forest, K-Nearest Neighbors, and Neural Networks. The accuracy levels achieved by these techniques are heterogeneous [27] and are always constrained by the amount and quality of data available.

From a practical perspective, it is clear that the healthcare industry still lacks many strong initiatives devoted to implementing AI-driven preventive clinical protocols with the intention of reducing the occurrence of MetS and its related NCD in the general population. In this context, the model proposed in this study adopted an innovative approach rooted in ‘Transformer’, a paradigm that has proven successful in large language models outside the healthcare field, and has become famous with the irruption of generative AI. By harnessing the contextual comprehension capabilities inherent in this architecture, novel connections between biomarkers, clinical data, and the NCD associated with MetS were extracted. Additionally, the proposed model has the potential to bring new products and business processes to the healthcare industry, such as product development strategies in telehealth solutions and create targeted marketing campaigns. The AI tool’s capabilities can be integrated into medical devices, such as wearable health monitors and smart scales, to provide real-time health data and feedback to users. Additionally, the development of AI-based tools necessitate high-performance computing capabilities and the demand of these resources can encourage further investment in such an advanced equipment, make it more available and portable. Industrial and technological partners can be the key contributors for providing pathways for advancing from validated prototypes into products.

Furthermore, at present, there is no specific programme for MetS prevention and the economic impact of this lack of public health policies is huge: the financial burden of the NCDs in the European Union reaches 25% of health spending and corresponds to a 2% loss of gross domestic product (GDP). Premature mortality from NCDs results in a loss of €115 billion per year to the economy, or 0.8% of EU GDP. Furthermore, the total cost of adult obesity in the EU was estimated at 70 billion Euro per year in 2016, including healthcare costs and lost productivity. In addition, about 7% of the national budgets across the EU is spent on NCD associated with obesity every year [28]. The absence of public health policies focused on MetS prevention results in high costs and inefficiencies. The project’s clinical validation of AI-based interventions provides grounded estimates of their effectiveness, which could substantially reduce MetS- and NCD-related complications. The proposed AI model can be a useful tool for early detection of high-prevalent NCD and to prioritize patients at high risk, thus allowing timely interventions which will result in the optimization of healthcare workflows and a significant reduction of healthcare costs. The model will allow the characterization of MetS risk and MetS-related complications in a personalized manner: by incorporating all the existing risk factors of MetS (including patient-related and socio-demographical factors) plus new biological biomarkers and accounting for gender, ethnic and geographical-specific variations. The AI analysis of patient data can provide valuable insights into MetS trends, risk factors, and treatment outcomes and can uncover patterns and correlations that may not be evident through traditional methods. Furthermore, novel biomarkers can be identified, allowing the development of new diagnostic and predictive tools, personalized phenotyping and development of precision medicine, as well as validate new hypothesis and identify potential drug targets.

By incorporating these insights into regular performance reviews, healthcare institutions can foster a culture of continuous improvement, ensuring that both hospital and individual provider performance are aligned with the goals of preventative care and patient well-being.

In summary, we have reported a model as a ‘proof-of-concept’ based on AI strategy capable of proactively helping the management of MetS and related NCD, with the potential of changing the current medical practice, in a more efficient and patient-centred manner, as explained above. This model can be deployed in various healthcare settings, including primary care clinics and remote telemedicine platforms, making MetS prevention more accessible to a broader population. Moreover, the scalability of AI allows it to handle large datasets and support population-wide preventive initiatives. The integration of the AI tool into existing healthcare systems or platforms will permit the generation of risk scores, assist both healthcare providers and individuals in the decision-making process, and give patients the option to actively engage in their own health management and track their progress while collaborating with valuable data collection using specific forms.

## Supplementary Material

ckaf098_Supplementary_Data

## Data Availability

Data is available upon request to the corresponding authors.
