# Supplementary figures and images for: A model based on artificial intelligence for the prediction, prevention and patient-centred approach for non-communicable diseases related to metabolic syndrome
Source: Eur J Public Health. 2025 Jul 3;35(4):642–9. doi: 10.1093/eurpub/ckaf098 (PMC12311355; doi:10.1093/eurpub/ckaf098)

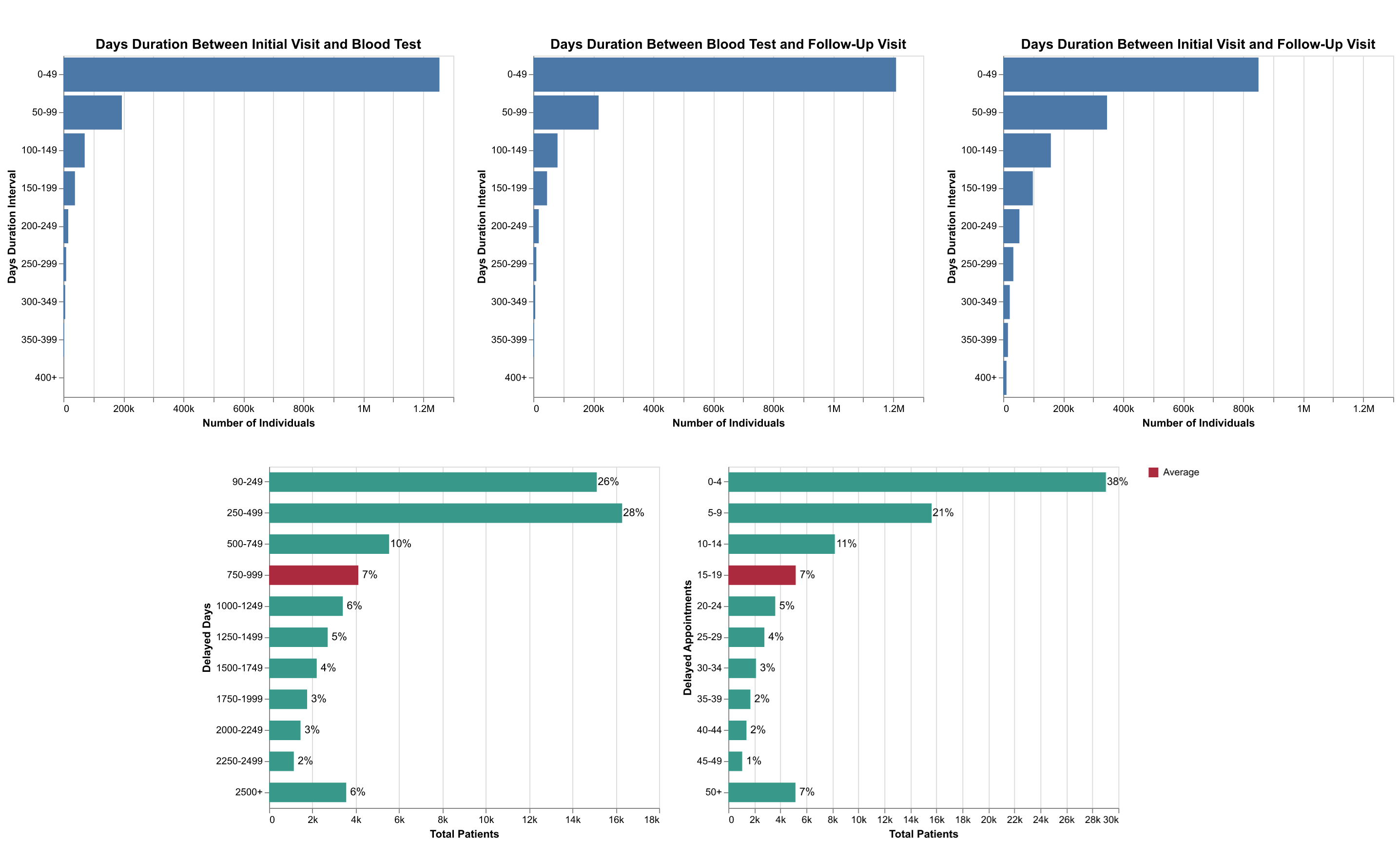

Supplement: ckaf098_Supplementary_Data [file ckaf098_supplementary_data.zip › ckaf098_Supplementary_Data/ejph-2024-11-om-0779-File005.tiff]

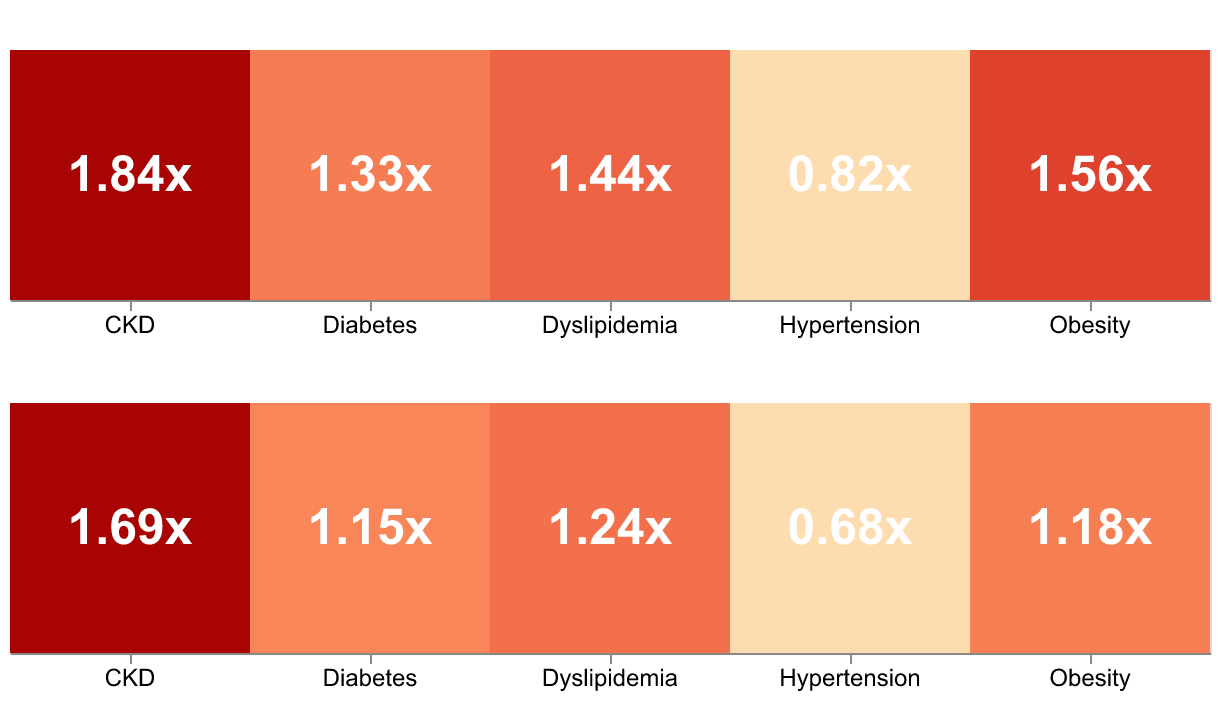

Supplement: ckaf098_Supplementary_Data [file ckaf098_supplementary_data.zip › ckaf098_Supplementary_Data/ejph-2024-11-om-0779-File006.tiff]
